# Zinc-bound metallothioneins and immune plasticity: lessons from very old mice and humans

**DOI:** 10.1186/1742-4933-4-7

**Published:** 2007-09-29

**Authors:** Eugenio Mocchegiani, Robertina Giacconi, Elisa Muti, Catia Cipriano, Laura Costarelli, Silvia Tesei, Nazzarena Gasparini, Marco Malavolta

**Affiliations:** 1 Immunology Ctr. (Section Nutrition, Immunity and Ageing) Res. Dept. I.N.R.C.A., Ancona, Italy

## Abstract

The capacity of the remodelling immune responses during stress (named immune plasticity) is fundamental to reach successful ageing. We herein report two pivotal experimental models in order to demonstrate the relevance of the immune plasticity in ageing and successful ageing. These two experimental models will be compared with the capacity in remodelling the immune response in human centenarians. With regard to experimental models, one model is represented by the circadian rhythms of immune responses, the other one is the immune responses during partial hepatectomy/liver regeneration (pHx). The latter is suggestive because it mimics the immunosenescence and chronic inflammation 48 h after partial hepatectomy in the young through the continuous production of IL-6, which is the main cause of immune plasticity lack in ageing. The constant production of IL-6 leads to abnormal increments of zinc-bound Metallothionein (MT), which is in turn unable in zinc release in ageing. As a consequence, low zinc ion bioavailability appears for thymic and extrathymic immune efficiency, in particular of liver NKT cells bearing TCR γδ. The remodelling during the circadian cycle and during pHx of zinc-bound MT confers the immune plasticity of liver NKT γδ cells and NK cells in young and very old mice, not in old mice. With regard to human centenarians and their capacity in remodelling the immune response with respect to elderly, these exceptional individuals display low zinc-bound MT associated with: **a) **satisfactory intracellular zinc ion availability, **b) **more capacity in zinc release by MT, **c) **less inflammation due to low gene expression of IL-6 receptor (gp130), **d) **increased levels of IFN-gamma and number of NKT cell bearing TCR γδ. Moreover, some polymorphisms for MT tested in PBMCs from human donors are related to successful ageing. In conclusion, zinc-bound MT homeostasis is fundamental to confer the immune plasticity that is a condition "sine qua non" to achieve healthy ageing and longevity.

## Background

Immune plasticity is a condition "sine qua non" for health ageing. The absence of the plasticity leads the organism to be a "low responder" to oxidative stress with subsequent appearance of age-related diseases. The remodelling of the immune system to various harmful stimuli allows a prompt immune response and the organism becomes a "high responder". Therefore, the capacity in the remodelling can be considered the plasticity of the immune system against the oxidative stress. The lack of this capacity leads the cells of the immune system to undergo to cell-death or necrosis triggered by oxidative stress [1,2]. Such a plasticity is a common event in young-adult age during transient stress-like conditions. During ageing, the capacity of the remodelling of the immune system is very limited because the stress-like condition is chronic [2]. This phenomenon allows reduced immune responses to oxidative stress and a low cellular capacity in DNA-repair [3]. As a consequence, the risk of the appearance of age-related diseases, i.e. cancer and infections, is high [3]. On the other hand, the "free radical theory", which takes into account the production of free radicals by oxidative stress, is the more common theory of the ageing process [4]. The molecular basis of the absence of the immune plasticity in ageing is poor understood and, at the same time, poor also studied up to ten years ago when the scientific community has seen an high increment of exceptional individuals, like centenarians, among elderly people. Indeed, healthy centenarians differ from "normal" aged individuals for their optimal metabolic compensation and immune response and for the ability to efficiently counter the alteration of the oxidative status typical of ageing. In this context, various hypotheses have been proposed in order to reach successful ageing. Limited inflammation, higher homing of stem cells to substitute the damaged cells, an increased capacity in DNA-repair and, finally, a major genomic integrity are characteristics of oldest individuals [5]. However, the capacity of the remodelling of the immune system can be also pivotal in these exceptional individuals and, as such, an improved immune plasticity. In this context, the role played by zinc and Metallothioneins (MT) may be crucial for the following reasons. First, zinc is a trace element indispensable for the efficiency of the immune system both in thymic and extrathymic T-cell pathways [6,7], and this latter is fundamental in order to compensate thymic failure in ageing [8]. Second, MT is relevant in zinc sequestering and in zinc release for the immune efficiency and antioxidant response during transient stress [9]. The zinc release by MT does not occur in ageing because stress-like condition is chronic leading to low zinc ion bioavailability for immune efficiency and for zinc-dependent biological functions, such as enzyme antioxidant activity and DNA-repair [10]. Third, the gene expression of MT is induced by pro-inflammatory cytokines (IL-1, IL-6 and TNF-α) during stress and inflammation [11]. In addition, it has been recently shown that IL-6 regulates the zinc transporter Zip14 in liver and contributes to the hypozincemia of the acute-phase response coupled with enhanced MTmRNA induction [12]. Therefore, the increment of these cytokines, especially of IL-6, in ageing leads to abnormal increase of MT associated with low intracellular zinc ion bioavailability and impaired immune response [10]. Consistent with these findings, zinc and MT homeostasis is crucial in conferring immune plasticity during ageing taking also into account that satisfactory zinc ion bioavailability is observed in human centenarians [1]. In the present paper, two relevant experimental models are reported in order to demonstrate the relevance of the immune plasticity in ageing: the variations of the immune functions **a) **during the circadian cycle and **b) **during the compensatory liver growth after partial hepatectomy. The choice of these two experimental models is based by previous findings showing the impact that the thymic circadian variations [13] and the liver extrathymic T-cell pathway [1] have in the economy of the immune response in ageing and successful ageing. In addition, the model of young partial hepatectomy/liver regeneration is very interesting because, other than a good model for the study of acute and chronic inflammation, it mimics the ageing process in thymic failure and in impaired peripheral immune efficiency at 48 hr after partial hepatectomy in young pHx mice [14]. Young, old and very old mice were used in both experimental models. A parallelism with elderly and nonagenarian/centenarians is reported with a special focus on some MT, IL-6 and TNF-alpha polymorphisms because related to successful ageing or to the appearance of age-related diseases in dependence of the genotype considered.

## Immune plasticity: model of the circadian cycle in mice

Young mice display fluctuating variations in plasma zinc and in thymic endocrine activity during the circadian cycle with nocturnal peaks. By contrast, no significant variations occur in old mice during the circadian cycle with an absence of nocturnal peaks [13]. This absence is also observed in peripheral immune efficiency. In particular, the low Natural Killer (NK) cell activity observed in old mice during the light period is also maintained during the dark with no significant variations during the whole circadian cycle [1,13,15]. Such a defect in old mice is closely related to the appearance of age-related diseases (cancer and infection) and subsequent death [13] (see Table 1). Conversely, immune peripheral variations occur in young-adult mice (Table 1) coupled with the capacity of young mice to respond to external antigenic stimuli and, subsequently in avoiding diseases triggered by the oxidative stress, via circadian variations of neuroendocrine mediators (cortisol, melatonin, insulin like growth factor) [16]. It has been shown that both IL-2 and IFN-γ, that are relevant for NK cell activity, display nocturnal peaks in young mice [1,17]. Nocturnal peaks of thymic and peripheral immune functions also occur in very old mice [1]. It has been also shown that the circadian variations of NK cells are under the control of a specific gene (*Period *2) [17] located in the suprachiasmatic nuclues and belonging to *Period *family genes [18]. In absence of these "clock genes", the innate immune variations are lost with the appearance of tumours [19]. Therefore, independently by the mechanism/s or genes involved, all these findings are clear evidences that the immune variations during the circadian cycle are fundamental in maintaining the immune efficiency and plasticity, which are in turn indispensable to achieve health longevity. In this context, an interesting aspect of the immune system, i.e. the liver extrathymic T-cell pathway deputed to compensate the thymic failure in ageing [8], shows variations during the circadian cycle in young and very old mice, but not in old ones [20]. The liver NKT cells bearing TCR αβ or γδ play an intriguing role. These cells are the first sentinels for the host defence against viruses and bacteria because secreting IL-2 and IFN-γ, which in turn affect the activity of classic NK cells [21]. These particular liver NKT cells display a circadian rhythm in young and very old mice with significant modifications between the light and dark period. In particular, the number of NKT γδ cells increases in young and very old mice during the dark, whereas it remains unmodified in old mice (Table 1). The number of NKT αβ cells displays an opposite trend with a decrement in young, old and very old mice during the dark as compared to the light period [15]. These findings suggest that NKT γδ cells may be more involved in the maintenance of liver extrathymic immune plasticity during ageing leading to a possible successful ageing. This maintenance may be due to a better preservation by cell death of NKT γδ cells than αβ because of low Fas expression (CD95) in NKT γδ cells in oldest individuals [22]. On the other hand, a significant decrement in liver NKT cells expressing Fas (CD95) occurs in very old mice in the dark as compared to old mice during the same period [20]. By contrast, old mice display lower number of NKT γδ cells for the whole circadian cycle, impaired NKT γδ cell cytotoxicity and decreased production of IL-2 and IFN-γ in comparison with very old mice [15] (Table 1). Thus, the functionality and the number of these cells, in particular of liver origin, are pivotal to reach successful ageing because some age-related diseases, such as infections, might be avoided. Indeed, old infected patients display a lower number of NKT γδ cells than elderly [23], giving further support to the relevance of liver NKT γδ cells for the host defence against viruses and bacteria [21].

**Table 1:** Some biological and immune parameters in young (A), old (B) and very old mice (C) during the circadian cycle and during partial hepatectomy/liver regeneration. A parallelism with old and centenarians is reported.

	**MICE**									**HUMANS**	
			
	**Circadian Cycle**				**Partial Hepatectomy**					**Old**	**Centenarians**
		*LP*	*DP*			*0 h*	*48 h*	*15 gg*			
			
**Number of NKTγδ cells**	*A*	++	+	**MT**	*A*	-	+++	-	**MT**	+	-
	*B*	--	--		*B*	+	+	+			
	*C*	+	++		*C*	-	+	-			
**NKT cell cytotoxicity**	*A*	++	+++	**NKT cell cytotoxicity**	*A*	++	-	++	**NKT cell cytotoxicity**	-	+
	*B*	--	--		*B*	-	-	-			
	*C*	+	++		*C*	+	-	+			

**IFNγ**	*A*	+	++	**Thymic endocrine activity**	*A*	++	-	++	**IFNγ**	-	+
	*B*	-	-		*B*	-	-	-			
	*C*	+	++		*C*	+	-	+			

**IL-2**	*A*	+	++	**IL-2**	*A*	++	-	++	**IL-6**	++	++
	*B*	-	-		*B*	-	-	-	**gp 130**	++	-
	*C*	+	++		*C*	+	-	+			

**Plasma zinc**	*A*	+	++	**Plasma Zinc**	*A*	++	-	++	**Plasma zinc**	-	+
	*B*	-	-		*B*	-	-	-			
	*C*	+	++		*C*	+	-	+			

+ = satisfactory ++ = high +++ = very high - = low -- = very low

LP = Light Period DP = Dark Period

## Immune plasticity: model of the partial hepatectomy/liver regeneration in mice

Partial hepatectomy/liver regeneration (pHx) is a good model for the study, other than the liver regeneration, of acute and chronic inflammation in ageing because of the likeness with ageing in impaired thymic endocrine activity, low zinc ion bioavailability and peripheral immune efficiency (NK cell activity and IL-2 production) in young pHx mice at 48 hr after pHx [14,24] (see Table 1). A complete remodelling of zinc ion bioavailability and immune efficiency however, occurs in the late period of compensatory liver growth (7^th ^and 15^th ^day) in young pHx mice. By contrast, no remodelling occurs in old mice displaying the same low zinc ion bioavailability and impaired immune functions for the whole period of the compensatory liver growth (time 0, 48 hr, 7^th ^and 15^th ^day) [14] (Table 1). These findings are intriguing because they suggest that pHx is also a good model in order to show the immune plasticity and, at the same time, the relevance of this plasticity in liver extrathymic T-cell pathway during ageing. This assumption is supported by the fact that very old pHx mice show the same pattern in zinc ion bioavailability, in liver NKT cell activity as well as in IL-2 production observed in young pHx mice [20] (Table 1). In other words, zinc ion bioavailability, liver NKT cell activity and IL-2 production are not lost during the compensatory liver growth in very old mice, but a remodelling occurs in the late period of the liver regeneration (15^th ^day), as occurring in young pHx mice [20]. These findings in very old mice, while on one hand demonstrate the presence of the immune plasticity in very old age, on the other hand they pinpoint that very old mice are still capable in responding to a great inflammation, like partial hepatectomy, with a remodelling of the liver immune efficiency.

This fact is very important in the oldest individuals because it means that many age-related diseases may be avoided in centenarians. As a consequence, very old individuals become "high responders" to oxidative stress and inflammation, as occurring in the young [1,25]. Indeed, the lack in responding to a great inflammation (like partial hepatectomy) in old age provokes a shorter survival in old pHx mice in comparison with old sham controls, because old pHx mice display a greater incidence of cancer and infections [24]. Thus, a good functioning of liver extrathymic immune plasticity is pivotal to reach successful ageing taking into account that liver extrathymic T-cell pathway is prominent in ageing [8].

## Immune plasticity: Lesson from Centenarian subjects

The study of human longevity and in particular the possibility to remodel many body homeostatic mechanisms, especially in remodelling some relevant immune functions, such NK cell cytotoxicity, in very old age may provide intriguing insights in the understanding why some organisms have the capacity to reach healthy ageing with respect to normal elderly. Centenarians represent the longevity phenotype naturally occurring and they are the best model because for the entire life a centenarian has taken place in an environment that continuously pushed the organism to cope with intrinsic and extrinsic antigenic loads. On the other hand, no other organism has experienced the rapid changes in hygiene, disease prevention, technology, and other areas as those that occurred to human populations in the last century, chiefly in developed countries. Therefore, the model of centenarians is not simply a good model to study the human longevity, with respect to well-studied animal models (see above), but also provides unique insights into the complex network of biological and non biological factors that guide individual survival at old age. In this contest, the capacity in remodelling the immune function (i.e. immune plasticity) is fundamental to reach centenarian age. Such an assumption is supported by some data on NKT γδ cells obtained in human centenarians during the light period. In these exceptional individuals, the major preservation of NKT γδ cells [22] is coupled with satisfactory NKT cell cytotoxicity and enhanced IFN-γ and IL-2 production [23,26] (Table 1). Such a preservation and capacity to maintain a satisfactory innate immune response is strictly related to the inflammatory status and genes related to inflammation. Among these genes, the MT homeostasis and the intracellular zinc ion bioavailability, as reported above, is fundamental in reducing inflammation with subsequent achievement of successful of ageing. Indeed, the analysis of some polymorphisms for the various isoforms of MT, mainly isoform I and II, confirms this assumption. Recent findings show a novel polymorphism of MT1A (in position +647 and with an aminoacid transition Asp/Asp in MT gene, called A/A genotype) involved in successful of ageing and coupled with lower levels of IL-6 and major intracellular free zinc ion availability [27]. In contrast, old subjects carrying an aminoacid transition Asp/Thr and Thr/Thr (called A/C and C/C genotypes, respectively) display higher levels of IL-6 and low zinc ion availability [27]. The same phenomenon also occurs for another polymorphism for MT1A in position -197. A significant increment of G+ genotype (GC and GG allele) occurs in nonagenarian-centenarian subjects as compared to old subjects carrying G- genotype (CC allele) suggesting a predisposition of G+ subjects to the longevity. Subjects carrying G+ genotype also provides less inflammation due to lower circulating levels of IL-6, low gene expression of MT and higher intracellular zinc ion bioavailability [E. Muti, unpublished results]. Although these findings are strongly of interest because these novel MT polymorphisms are involved in longevity, some other genes related to the inflammation are also involved in longevity. The most relevant is the polymorphism of IL-6. In this context, the polymorphism for a C to G transition at nucleotide -174 of the IL-6 gene promoter (-174 C/G locus) shows increased IL-6 production in C-(GG genotype) but not in C+ (CC and CG genotypes) old subjects, and this phenomenon was significant only in males [28,29]. Subsequently, it has been found that C- subjects show also high MT and low intracellular zinc ion bioavailability and impaired innate immune response [29]. Moreover, IL-6 -174 C/G polymorphism is an independent predictor of cardiovascular death after an acute coronary syndrome (ACS) in male patients [30] as well as in atherosclerosis worsening in old people (29). ACS patients carrying the IL-6-174 C-(GG) genotypes underwent a marked increase in 1-year follow-up mortality rate (HR = 3.89, 95% CI 1.71–8.86, *P *= 0.001), thus suggesting that the IL-6 -174 C/G polymorphism can be added to other clinical markers in order to identify a subgroup of elderly ACS male patients at a higher risk of death [30]. On the other hand, ACS as well as some other cardiovascular diseases (stroke and CAD) display high MT and high inflammatory status coupled to low intracellular zinc ion bioavailability and impaired innate immunity [31]. Another intriguing finding is the association with longevity of alleles of IL-10 and TNF-alpha known to have opposite functions in inflammatory reactions, IL-10 acting predominantly as an anti-inflammatory and TNF-alpha as a pro-inflammatory factor. The combination of IL-10 and TNF-alpha genotypes shows that there is a significant increase of the "anti-inflammatory" (IL-10 -1082GG/TNF-alpha -308GG) genotype (named A- subjects) in centenarian men over controls [32]. This finding is in agreement with the recent discovery showing instead old individuals carrying TNF-alpha -308 AA genotype (named A+ subjects) more involved in infection relapses coupled with low zinc intracellular zinc ion bioavailability, increased MT, diminished IL-10 and impaired innate immunity [33]. All these data suggest on one hand that some polymorphisms of MT may be involved in longevity, on the other hand that some other polymorphisms related to inflammation (IL-6 and TNF-alpha) are involved in the appearance of some age-related diseases but, at the same time, these genes are strictly related to MT homeostasis, zinc ion availability and immune plasticity. Therefore, the MT gene may be the core of the complex genetic network that regulates the inflammatory status and, consequently, the longevity or the appearance of age-related diseases. Indeed, some other MT polymorphisms in position -209 (A/G genotype) is mainly involved in atherosclerosis worsening and diabetes type II [34].

## Mechanisms of action in maintaining the immune plasticity

**Figure 1:**
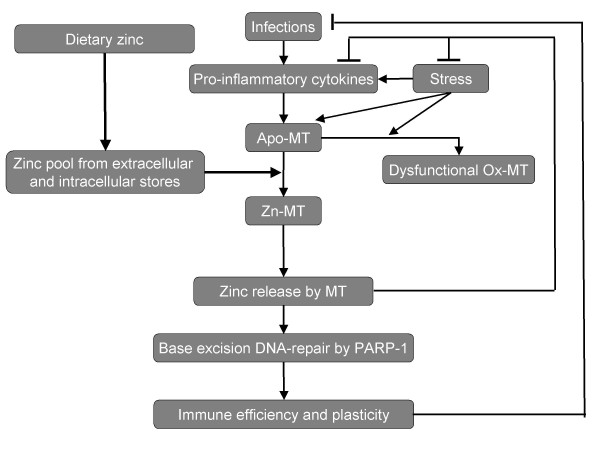
Schematic mechanism of the interplay between Metallothionein (MT), inflammation and stress. Infections and stress trigger an inflammatory response by increasing the production of pro-inflammatory cytokines which, in turn, stimulate the gene expression of Metallothioneins (which are produced as apo-MT). These proteins need zinc to properly absolve their function of zinc "releasers" during stressing condition. The zinc signal produced by release of zinc from Zn-MT is necessary to activate zinc dependent antioxidant and repairing enzymes, such as PARP-1, thus contributing to down-regulate stress and inflammation. The zinc pool which act as Zn++ reservoir for Zn-MT, is strictly dependent upon the dietary intake of this trace element, so that also dietary habits also contributes along with the genetic background to immune efficiency and plasticity.

It has been demonstrated that the zinc ion bioavailability is fundamental for the efficiency of the immune system [6,7]. The loss of zinc ions by intestinal malabsorption or by reduced food intake provokes a zinc deficiency with damage in cell-mediated immunity, including thymic efficiency, NK cell activity, and cytokine production [35]. In particular, during zinc deficiency some cytokines, such as IL-2, IL-12, IFN-α, IFN-γ, decrease other cytokines, such as TNF-α, IL-1, IL-6, increase [36]. In this context, zinc more affects the cytokine production by Thl than Th2 cells [37]. That zinc has a beneficial effect on IFN-α production by Th1 cells is supported by the discovery in virus transfected cells showing a protein Staf-50 involved in a new family of IFN-α production that contains two zinc finger motifs [38]. More recently, zinc potentiates the antiviral action of IFN-α tenfold [39]. These findings suggest an unbalance of Thl/Th2 paradigm during zinc deficiency towards Th2 cytokine production [6,37], which leads to the induction of some proteins deputed in fighting the oxidative stress. In this context, metallothionein (MT) plays a pivotal role because it sequesters and dispenses zinc [40]. MT acts as antioxidant against wide spectrum of stressor agents, because zinc-sulfur cluster is sensitive to changes of cellular redox state and oxidizing sites in MT (reduced number of thiol groups) induce the transfer of zinc from its binding sites in MT to those of lower affinity in other proteins [9,41]. This transfer occurs in conferring biological activity to antioxidant metalloenzymes, such as superoxide dismutase, in the base excision DNA-repair by PARP-1, in the genomic stability by telomerases, and, finally, in conferring directly or indirectly, via zinc finger motifs, the immune efficiency [10] (Fig. 1). Therefore, the redox properties of MT and their effect on zinc in the clusters are crucial for the biological functions of MT. Indeed, MT is peculiar in cellular proliferation and in protecting cells against cytotoxic effects of reactive oxygen species, ionizing radiations, electrophilic anti cancer drugs, mutagens and heavy metals [9]. A peculiar role of MT is played during partial hepatectomy/liver regeneration, with a strong MT induction that is useful, other than in facilitating the liver regeneration by various hepatocyte growth factors, in protecting the cells by the inflammation after partial hepatectomy. High MT, either as gene expression or protein, is present in young pHx mice at 48 h from pHx coupled with low zinc ion bioavailability, high IL-6 and impaired thymic and extrathymic T-cell pathways [14,24]. A complete down-regulation of MT and IL-6 followed by a restoration of the immune efficiency occurs in the late period of the compensatory liver growth (7^th ^and 15^th ^day from pHx) [24]. By contrast, the high MT and IL-6 gene expressions as well as the low zinc ion bioavailability and the impaired immune functions, already present in old mice, are not modified during the liver regeneration in old pHx mice. An intriguing aspect is the complete remodeling of MT, zinc ion bioavailability and immune function in very old pHx mice at 7^th ^and 15^th ^day from partial hepatectomy [24]. These findings further demonstrate that MT is not protective against chronic inflammation, like in ageing, because it is unable in release zinc, whereas its protective role occurs in young-adult age [1]. Therefore, MT turns from role of protection in young age to harmful one in ageing due to its inability in zinc release [1]. This phenomenon in ageing provokes low zinc ion bioavailability for zinc-dependent enzyme antioxidant activity, for base excision DNA-repair by PARP-1, and for thymic and extrathymic T-cell pathways. Therefore, MT follows the "Antagonistic Pleiotropy Theory of Ageing" and plays a pivotal role in zinc turnover in ageing and consequently in conferring the immune plasticity. Such an assumption is supported during the circadian cycle in which the high nocturnal peaks of zinc and immune efficiency observed in young and very old mice are related to low MT either as gene expression or as protein [1]. No circadian variation of MT occurs in old mice [1]. In addition, low MT gene expression and good zinc ion bioavailability are also observed in lymphocytes from centenarians [1]. This phenomenon of MT in regulating zinc turnover is closely dependent by the inflammatory status, in particular by the gene expression and induction of pro-inflammatory cytokines, such as IL-6, and of its sub-unit receptor gp130. Although, IL-6 can elicit pro-inflammatory or anti-inflammatory effects depending on the in vivo environmental circumstances [42], abnormal enhanced production of IL-6 is an index of great inflammation and disability in ageing [43]. Also gp130 gene expression is constantly high in ageing [44] leading to continuous increase of MT followed by the stealing of intracellular zinc ions and no subsequent zinc release by MT. As a consequence, low zinc ion bioavailability appears in the maintenance of the immune plasticity in ageing [1]. It is not a simple coincidence that both very old mice and centenarians display low gp130 despite IL-6 is high (44). This fact allows low MT induction, good zinc ion bioavailability, satisfactory immune efficiency and an increased capacity in base excision DNA repair by PARP-1 in very old age (mice and humans). By contrast, high MT, IL-6 and gp130 coupled with reduced capacity in base excision DNA repair are present in elderly and in old infected patients [23]. In these latter, alterations in DNA-repair and in MT are still more severe. Indeed, abnormal high expression of MT is an index of unfavourable prognosis in cancer and infections [45]. Therefore, zinc-bound MT homeostasis, via IL-6 and gp130, is a fundamental mechanism in conferring the immune plasticity in order to reach successful ageing. MT can be thus considered a potential biological and genetic marker of immunosenescence upstream affecting functional biochemical cascade involved in the maintenance of the immune plasticity, in particular liver NKT γδ cells, with subsequent successful ageing.

## Conclusion and future remarks

In conclusion, the zinc-bound MT homeostasis may be considered a possible genetic marker of immunosenescence affecting the immune plasticity and consequently the achievement of successful ageing with a pleiotropic role during the life: protective in young adult age and harmful in elderly. This fact is evident in both experimental models herein described in order to show the relevance of MT homeostasis in conferring the immune plasticity in very old age. However, an intense debate has arisen during the last decade around the possible dangerous or protective role of MT in old and very old age. In fact, in contrast with their pleiotropic role, as suggest above, some authors have found also evidence that MT over-expression may be linked to enhanced survival and protection against oxidative damage [46,47]. An intriguing hypothesis to resolve this matter, is proposed for the first time in this manuscript, regarding to the possible interplay between the nutritional status and MT expression. The hypothesis is that MT overexpression may be dangerous and thus linked to shorter a dysregulated immune response and short survival when the nutritional status for zinc is impaired due to low dietary intake of this trace element or to malabsorption, which in turn are common features in elderly subjects [37]. The reasons to support this hypothesis arise from the well known capability of intracellular free zinc ion to suppress inflammation down-regulating the production of TNF-alpha [48]. So that when the immune inflammatory response has to be suppressed, free zinc ions are released from MT to down-regulate the production of TNF-alpha. However, in presence of an impaired zinc status, during an inflammatory processes an high expression of MT might be responsible of delaying the anti-inflammatory pathway through sequestration of free zinc ions. In addition, most of the MT might be oxidized and their capability to produce an intracellular anti-inflammatory zinc signal via NO-induced release might be also impaired. Conversely, in presence of an adequate zinc status, even if MT may be highly expressed, their capability to release intracellular free zinc ions to activate the anti-inflammatory pathway might be increased, with a consequent rapid down-regulation of TNF-alpha. A strong support to this hypothesis arise from experiments on the influence of zinc deprivation or supplementation on TNF-induced lethality in MT-null mice compared to wild type [49]. In both mice strains, zinc deprivation increased the susceptibility to TNF toxicity, but surprisingly zinc deprived MT-null mice were more resistant than zinc deprived wt mice. Conversely, zinc supplementation displayed overall protective effects. A further support to this hypothesis is that zinc supplementation in elderly subjects can increase MT levels but decrease at the same time inflammatory markers [E. Mocchegiani et al., preliminary results from Zincage Project, unpublished], and that optimal nutrition, including an adequate intake of zinc, promotes functional health status, mental well associated to reduced mortality [50,51]. In other words, MT homeostasis might be the connecting link between environmental/genetic factor in determining the longevity. Therefore, the capacity in remodelling immune functions (immune plasticity), via MT homeostasis combined with an adequate intake of zinc during the whole life, may represent peculiar characteristics of those exceptional individuals who reach the extreme limit of lifespan.
